# Hydrodynamic Simulation and Experiment of a Self-Adaptive Amphibious Robot Driven by Tracks and Bionic Fins

**DOI:** 10.3390/biomimetics9100580

**Published:** 2024-09-24

**Authors:** Minghai Xia, Qunwei Zhu, Qian Yin, Zhongyue Lu, Yiming Zhu, Zirong Luo

**Affiliations:** 1 College of Intelligence Science and Technology, National University of Defense Technology, Changsha 410073, China; xiaminghai@nudt.edu.cn (M.X.); zhuqunwei@nudt.edu.cn (Q.Z.); luzhongyue@nudt.edu.cn (Z.L.);; 2 College of Energy and Power Engineering, Changsha University of Science and Technology, Changsha 410076, China; yqziqiang@mail.sdu.edu.cn

**Keywords:** amphibious robot, undulating fin, compound drive, computational fluid dynamics, track robot, hydrodynamic simulation

## Abstract

Amphibious robots have broad prospects in the fields of industry, defense, and transportation. To improve the propulsion performance and reduce operation complexity, a novel bionic amphibious robot, namely AmphiFinbot-II, is presented in this paper. The swimming and walking components adopt a compound drive mechanism, enabling simultaneous control for the rotation of the track and the wave-like motion of the undulating fin. The robot employs different propulsion methods but utilizes the same operation strategy, eliminating the need for mode switching. The structure and the locomotion principle are introduced. The performance of the robot in different motion patterns was analyzed via computational fluid dynamics simulation. The simulation results verified the feasibility of the wave-like swimming mechanism. Physical experiments were conducted for both land and underwater motion, and the results were consistent with the simulation regulation. Both the underwater linear and angular velocity were proportional to the undulating frequency. The robot’s maximum linear speed and steering speed on land were 2.26 m/s (2.79 BL/s) and 442°/s, respectively, while the maximum speeds underwater were 0.54 m/s (0.67 BL/s) and 84°/s, respectively. The research findings indicate that the robot possesses outstanding amphibious motion capabilities and a simplistic yet unified control approach, thereby validating the feasibility of the robot’s design scheme, and offering a novel concept for the development of high-performance and self-contained amphibious robots.

## 1. Introduction

Natural evolution has rendered biological systems far more advanced than many of the capabilities of modern technology [1]. From the observation of nature and its various biological process, researchers were inspired to design efficient bioinspired systems to solve a wild range of problems in a low-cost and sustainable way [2]. Recent innovations of bioinspired advanced systems have led to the development of biomimetic robotics, including bioinspired structures, materials, actuators, sensors, and control methods.

As an important branch, biomimetic amphibious robots have been attracting increasing attention. Compared with conventional mobile robots that are capable of functioning only in a single medium, amphibious robots exhibit remarkable advantages such as multi-terrain motion maneuverability [3], environmental adaptivity [4], and obstacle climbing capacity [5]. Due to these benefits, amphibious robots hold broad prospects in the fields of industry, defense, and transportation [6]. For example, they can be deployed in scenarios such as environment protection [7], resource exploration [8], and search and rescue [9].

Traditional amphibious robots simply integrate propellers and tracks or wheels to realize aquatic and terrestrial locomotion [10,11]. However, the robots are required to switch among different propulsion mechanisms, resulting in a complex control system. Furthermore, the propellers are fragile in muddy and grassy areas, which renders them unsuitable for unstructured environments. In nature, there exist numerous creatures possessing outstanding amphibious locomotion capabilities, and researchers have drawn inspiration from amphibians. The last two decades have witnessed the advancement of bionic amphibious robots, including bionic crabs [12], turtles [13], lobsters [14], beavers [15], frogs [16], snakes [17], and salamanders [18].

Based on the driving mechanism, the existing bionic amphibious robots can be classified into single bionic amphibious robots and compound amphibious robots [6]. Single bionic amphibious robots imitate natural amphibians’ organisms and use the same propulsion components for land and underwater locomotion. This category encompasses three types of robots: snake-like, legged, and fish-like. The robotic snake uses the serpentine movement of its body to achieve propulsion on land and underwater [19,20]. However, the bionic robot snake lags far behind in terms of agility and speed when compared to real snakes, even with the assistance of passive wheels. The legged configuration represents a more comprehensive scheme for amphibious robots. Zhang et al. proposed a hexapod amphibious robot, namely AmphiHex-I [21]. The robot is actuated by six reconfigurable legs that can be controlled to switch between C-shaped legs and straight paddles for land and underwater motion, respectively. However, each leg should be equipped with a driven motor for transformation. In the updated version, AmphiHex-II [22], the robot adopts a variable-stiffness leg for lightweight design. The legs are constructed with rigid fan-shaped frames, working as walking legs, and protect the inner flexible flippers, which propel the robot underwater.

Some researchers used smart materials to build amphibious actuators. Zou et al. used a shape-memory polymer and an elastomeric actuator for reconfigurable limbs in a fin-based amphibious robot. The main problem was that the legs should have been transformed manually and reassembled to the robot [23]. Baines et al. developed a bionic turtle robot based on morphing limbs, which could change stiffness and cross-section area and adopted multiple walking and swimming gaits to adapt to different environments [24]. Wu et al. designed a 3D-printed tortoise-inspired soft robot. With four pneumatic bionic legs capable of bending in three dimensions, the robot could achieve six different gaits [25]. Although soft amphibious robots possess the merits of significant deformation and high adaptability, they are still at a considerable distance from practical applications.

In 2017, the American company issued Velox robot (Pliant Energy Systems, Brooklyn, USA) [26], which features a pair of ribbon fins for multi-terrain locomotion. The robot can adapt to various terrains, including ice, sand, beach, water, and seabed via the unified propulsion of undulating fins. Although similar works were conducted for optimal design [27], the undulating fin was relatively slow for land locomotion.

The compound amphibious robot combines various driving methods to meet practical requirements, showing a better balance between terrestrial and aquatic locomotion performance. Wang et al. [28] designed a leg–fin hybrid crab-like hexapod robot, which integrated two-tail swimming fins for underwater paddle flapping. AQUA2, which was developed based on the RHex series robot [29], adopted wheel–fin hybrid legs for amphibious propulsion [30]. The legs utilized a cage-like circular enclosure to protect the inner flippers when moving on the ground, and the cage worked like a wheel. The maximum velocity on land reached 0.9 m/s. Ma et al. proposed a hybrid amphibious robot named SHOALBOT [31], which could carry out multimode operation using a propeller-leg integrated propulsion device. Under low-speed mode, the robot could run as fast as three body lengths/s on land. When underwater, the robot body could be folded and the four propellers provided vectored thrust force to realize multi-DOF swimming. Yu et al. developed a wheel–leg–fin hybrid amphibious fish that could swim via dolphin-like and fish-like oscillations of its multi-joint body, and move fast on land via a wheel–propeller–fin mechanism [32].

From the above analysis, although the single-bionic-drive method does not require switching of propulsion mechanisms, it tends to perform better in only one environment at the expense of locomotion ability in another medium. The compound amphibious robot adopts optimal schemes for land and underwater scenarios, ensuring good propulsion performance in the two media. Nevertheless, due to the integration of distinct propulsion mechanisms, a greater number of drives are needed. More significantly, the robot must switch control systems, which enhances the difficulty of control.

In this paper, we proposed a track–fin-compound-driven amphibious robot, namely AmphiFinbot-II. The robot depends on bilateral undulating fins for underwater propulsion and tracks for land propulsion, demonstrating remarkable motion performance in both environments. The undulating fins and tracks share a common driving source, thereby moving synchronously. The robot is self-adaptive as it does not need to switch drive sources. The robot possesses identical control logics both underwater and on land, such as forward and backward movement, in-place rotation, and steering motion. Therefore, the same control system can be employed. In this paper, we concentrate on verifying the underwater propulsion performance of the robot through the computational fluid dynamics (CFD) method, and validate the amphibious motion function of the robot via experiments.

The rest of the paper is organized as follows: Section 2 introduces the structure design and locomotion principle of the amphibious robot. Section 3 establishes the simulation model and presents the hydrodynamic performance simulation of the robot. In Section 4, locomotion experiments are carried out in terrestrial and aquatic environments. A conclusion is drawn in Section 5.

## 2. Locomotion Principle

### 2.1. Robot Structure

**Figure 1:**
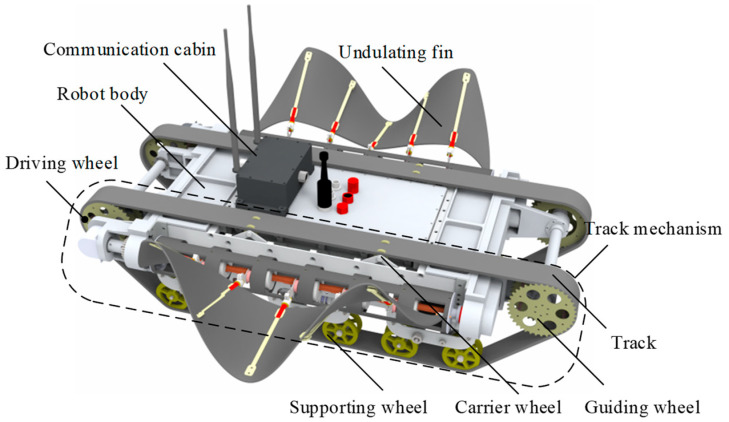
The overall structure of AmphiFinbot-II.

The overall design scheme of AmphiFinbot-II is presented in Figure 1, which consists of a robot body, undulating fins, a track mechanism, and a mobile communication cabin. The features of the robot are as follows: (1) Land driving is achieved through the track mechanism, featuring high speed, excellent maneuverability, and terrain adaptability. (2) Underwater driving is driven by bionic undulating fins, which are safer compared with propellers and can adapt to complex environments. (3) When the robot moves on a riverbed or in a water–land transition zone, the tracks and the undulating fins collaborate to provide thrust. (4) The robot body is designed as a rigid structure, facilitating the installation of sensors and manipulators. (5) Remote control is achieved by a movable sealing cabin. When the robot operates on land and in shallow water, the communication cabin is installed on the robot, and the robot employs wireless communication. When the robot is required to carry out deep-water operations, the communication cabin can be placed on the land and then connected to the robot through cables, and the robot utilizes wired communication. (6) The robot is compact and easily portable. We establish the robot’s dimensions based on the optimal space for manual operation, incorporating two handles at either end for convenient handling. While a higher chassis enhances obstacle navigation capabilities, it also results in an elevated center of gravity and increased hydrodynamic resistance. Consequently, the final design specifications for the robot are 0.81 m × 0.68 m × 0.22 m.

Despite the utilization of two sets of propulsion mechanisms, through the gear transmission system, the track mechanism and the undulating fin share a common driving source, enabling simultaneous control for the continuous rotation of the track and the wave-like motion of the undulating fin. Through this design, the robot employs different propulsion methods on land and underwater for optimal performance but utilizes the same drive source, eliminating the need for switching propulsion mechanisms.

**Figure 2:**
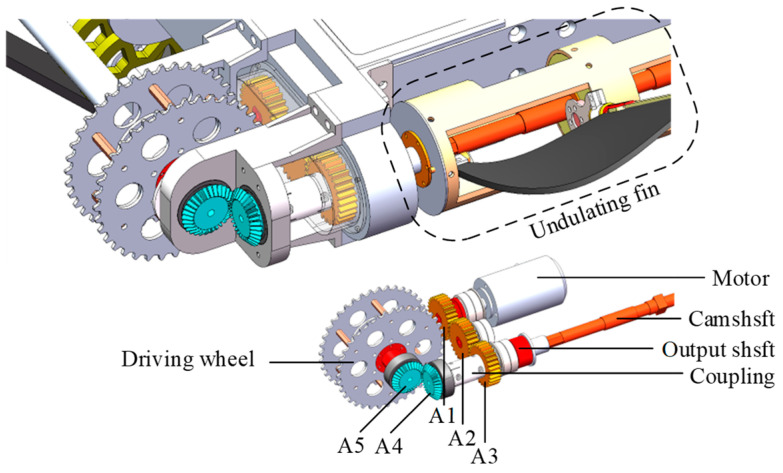
The track–fin compound drive mechanism.

The schematic diagram of the compound drive is depicted in Figure 2. The working principle is as follows. The motor’s output shaft is linked to spur gear A1, and the power is conveyed to the output shaft via spur gears A2 and A3. One end of the output shaft is connected with the undulating fin, which is responsible for generating underwater propulsion force via the wave motion. The other end of the output shaft is coupled to bevel gear A4 and drives vertically installed bevel gear A5 to rotate continuously. The track wheel, which is fixed with bevel gear A5, serves as the driving wheel, and actuates the track, the support wheel, and the guide wheel to rotate, thereby forming a complete-track walking mechanism.

**Figure 3:**
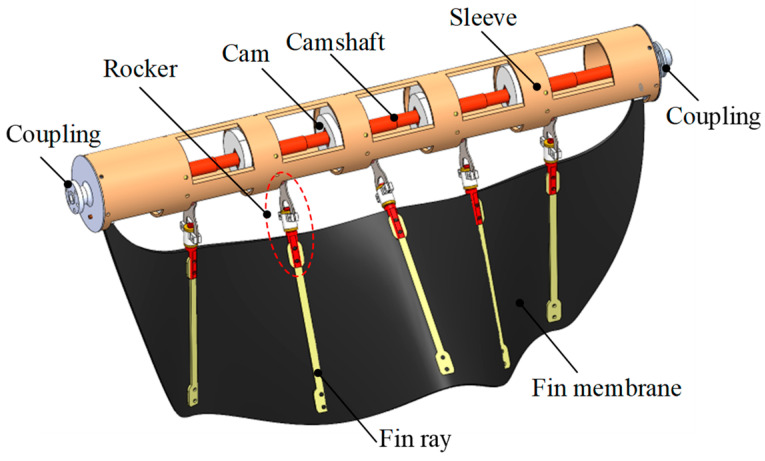
The bionic undulating fin structure.

The principle of the modular bionic undulating fin is depicted in Figure 3. Currently, the prevalent approach for undulating-fin-driven robots is to equip each oscillating joint with a drive motor [33,34,35], which offers the benefit of flexible adjustment of parameters such as wavelength, amplitude, and wave frequency. However, it suffers from several drawbacks, including a large number of motors, complex control, and difficult sealing. More crucially, due to the speed limit of the motor response, the wave frequency cannot be enhanced, and the maximum wave frequency can only reach 3–4 Hz [36]. Hence, we adopt a centralized drive scheme, where a motor drives the camshaft to rotate continuously. The cams connected in parallel to the camshaft installation have a 90-degree phase difference, converting the continuous rotation of the cams into the reciprocating swing of the pendulum rods. Since the swings of the adjacent pendulum rods always have a 90-degree phase difference, a waveform can be formed automatically. By controlling the direction of the camshaft, the transmission direction of the wave can be regulated. Although the waveform of this method is fixed and only the wave frequency and wave deflection angle can be adjusted, its advantages lie in its compact structure, simple control, and reliable sealing, so it is more suitable for field amphibious robots.

**Figure 4:**
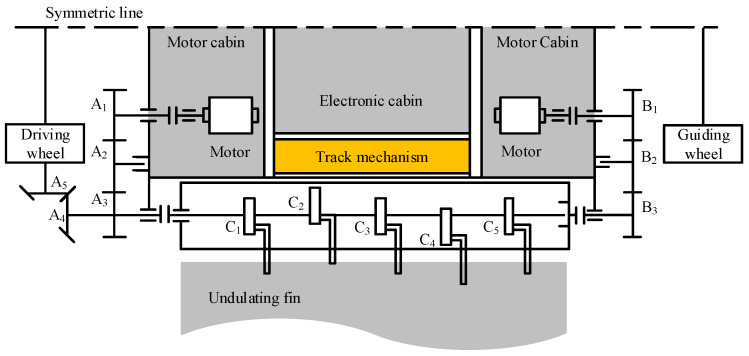
The layout and the transmission principle of AmphiFinBot-II.

The overall layout and transmission principle of the robot are depicted in Figure 4, which shows only half of the robot due to symmetry. As explained previously, a speed-regulating motor on the left side is applied for driving the track and the undulating fin. We configure another motor on the right side of the robot, which is also connected to the undulating fin via the gear drive (B1, B2, B3) to control the overall tilting angle of the fin for adjusting the robot’s attitude. The robot body is divided into three sealing cabins, i.e., the two motor cabins and the electrical cabin. This design separates the dynamic seal from the static seal to enhance the waterproof performance of the robot.

### 2.2. United Operation Mechanism

**Figure 5:**
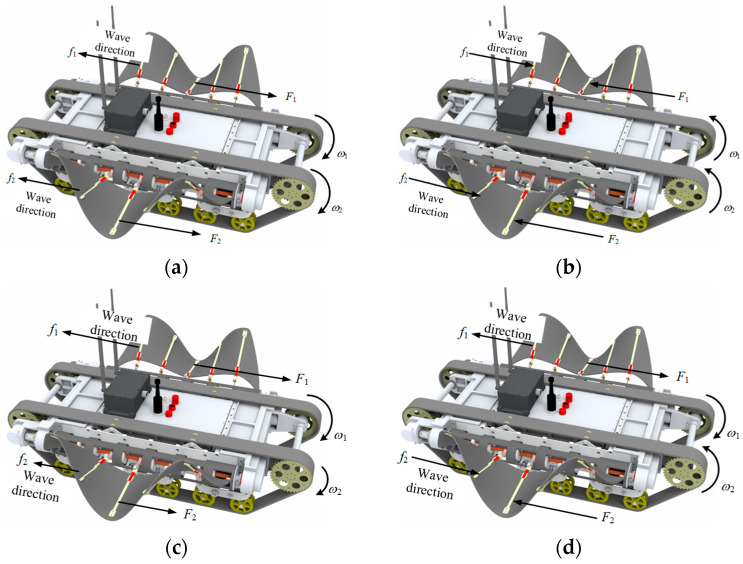
The united operation method of the robot in terrestrial and aquatic environments: (**a**) linear forward motion; (**b**) linear backward motion; (**c**) yaw motion; (**d**) rotation motion.

The existing hybrid amphibious robots operate in underwater, land and land transition environments, often involving distinct moving components and motion mechanisms, thereby necessitating a drive mechanism switch. AmphiFinbot-II employs the same drive source for undulating fins and tracks, making its motion compound and simultaneous. Moreover, the bilateral fins and tracks possess similar differential steering logics, thereby providing AmphiFinBot-II the same maneuvering strategy both underwater and on land, which implies that the robot does not require a switch of mechanical and control systems. The rotational speeds of the left and right tracks are defined as $\omega_{1}$ and $\omega_{2}$, with clockwise being positive. The frequencies of the undulating fins are $f_{1}$ and $f_{2}$, and the underwater propulsion forces generated are $F_{1}$ and $F_{2}$. The regulations for the robot conducting forward, backward, yaw and in situ steering actions are depicted in Figure 5.

- Forward movement: When the tracks on both sides rotate clockwise at the same speed ($\omega_{1}=\omega_{2}>0$), both tracks offer the same forward speed. Simultaneously, the left and right undulating fins transmit waves to the rear direction with the same frequency ($f_{1}=f_{2}>0$), and the undulating fins generate equal forward propulsion force ($F_{1}=F_{2}>0$). If underwater, the undulating fins propel the robot to move forward; if on the ground, the tracks drive the robot to move forward.
- Backward movement: As shown in Figure 5b, when the tracks on both sides rotate clockwise at the same speed ($\omega_{1}=\omega_{2}<0$), the undulating fins transmit waves to the front direction with the same frequency ($f_{1}=f_{2}<0$), and the propulsion force is negative ($F_{1}=F_{2}<0$). The robot moves backwards either underwater or on land.
- Yaw motion: Yaw motion can be achieved by regulating the differential rotation of the tracks in the same direction. For example, when $\omega_{1}>\omega_{2}>0$, the robot will turn to the right when moving forward on land. The left propulsive force is greater than that on the right side ($F_{1}>F_{2}>0$ ), and the combined force generates additional yaw torque, causing the robot to turn to the right underwater. Similarly, when $\omega_{2}>\omega_{1}>0$, the robot performs forward and left-turning locomotion. Due to symmetry, the robot also adopts the same left and right turn control methods when moving backward.
- Rotation motion: Turning in place is a special case of yaw motion, when the two-side tracks’ speeds are equal but for opposite directions. As shown in Figure 5d, when $\omega_{1}=-\omega_{2}>0$, there is $f_{1}=-f_{2}>0$ for undulating fins, so the resultant force is zero. The robot will carry out right-turning motion either on land or underwater. When $\omega_{1}=-\omega_{2}<0$, the robot will rotate left.

## 3. Hydrodynamic Simulation

### 3.1. Simulation Model

To verify the design feasibility and motion capability of the amphibious robot, the force and motion of the robot in the flow field under various control inputs were analyzed through computational fluid simulation (CFD).

**Figure 6:**
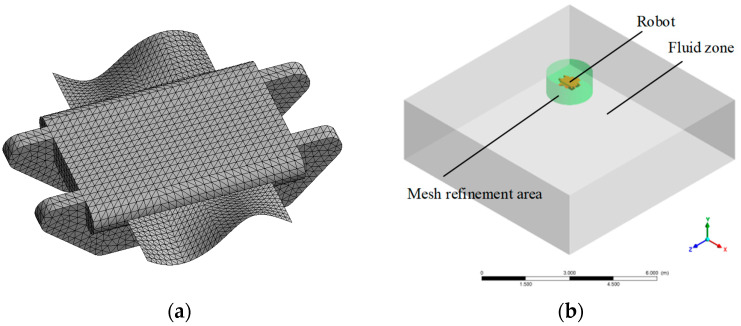
CFD simulation model: (**a**) the mesh model of the robot; (**b**) the calculation zone.

To simplify the grid division and accelerate the simulation calculation, the local particulars of the robot were streamlined. The simulation model was established, as depicted in Figure 6a, and the fluid domain is shown in Figure 6b. The grid of the outer fluid zone was coarsely divided; the robot and the adjacent local area which was shown in color were encrypted. According to the physical prototype’s design parameters, the simulation parameters of the robot are given in Table 1.

### 3.2. Mathematical Model

#### 3.2.1. Kinematic Model

**Figure 7:**
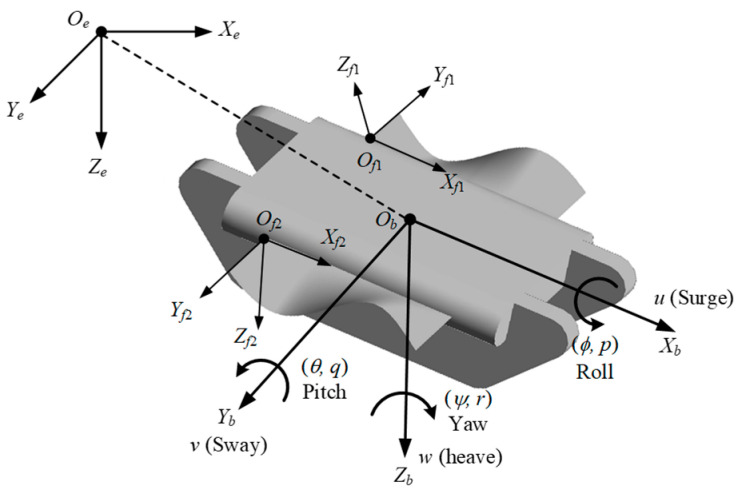
Coordinate system of the robot.

To describe the motion process of the amphibious robot in the flow field precisely, the kinematic model of the robot and the motion equation of the undulating fin were constructed. The coordinate system during the robot’s locomotion was defined, as shown in Figure 7.

- Global coordinate system, $O_{e}X_{e}Y_{e}Z_{e}$: The origin of coordinate $O_{e}$ was defined by taking any point within the earth; $O_{e}X_{e}$ pointed at any horizontal direction, $O_{e}Z_{e}$ was vertically downward to the ground, and the direction of $O_{e}Y_{e}$ was in accordance with the right-hand rule.
- Local coordinate system, $O_{b}X_{b}Y_{b}Z_{b}$: The origin $O_{b}$ was defined as the mass center of the robot. $O_{b}X_{b}$ was along the longitudinal axial direction. $O_{b}Z_{b}$ pointed straight down the body. $O_{b}Y_{b}$ was located to the right of the body.
- The fin coordinate system, *O_f_X_f_Y_f_Z_f_*: The origin $O_{f}$ was located at the base point of the undulating fin. $O_{f}X_{f}$ pointed forward along the undulating fin’s base line. $O_{f}Z_{f}$ was in the symmetrical plane of the undulating fin and pointed outwards. $O_{f}Y_{f}$ was determined by the right-hand rule. The coordinate systems $O_{f1}X_{f1}Y_{f1}Z_{f1}$ and $O_{f2}X_{f2}Y_{f2}Z_{f2}$ were established for the left and right undulating fins, respectively.

Suppose $\eta ={\left(X,Y,Z,\phi ,\theta ,\psi \right)}^{T}$ is the position and attitude vector of the robot in the global coordinate system. $\eta_{1}={\left(X,Y,Z\right)}^{T}$ is the three-dimensional coordinate, and $\eta_{2}={\left(\phi ,\theta ,\psi \right)}^{T}$ is the three-axis attitude angle, corresponding to roll, pitch, and yaw angle. $V={\left(u,v,w\right)}^{T}$ and $\omega ={\left(p,q,r\right)}^{T}$ are the three-axis linear and angular velocities of the robot in the local coordinate system, respectively. The relationship between the local coordinate system and the robot system can be expressed as follows:

(1) $$\{\begin{array}{c} {\dot{\eta }}_{1}=J_{1}\left(\eta \right)\cdot V,\\ {\dot{\eta }}_{2}=J_{2}\left(\eta \right)\cdot \omega , \end{array}$$

where $J_{1}\left(\eta \right)$ and $J_{2}\left(\eta \right)$ are the rotation matrices, which are solved as follows:

(2) $$J_{1}\left(\eta \right)=\left[\begin{array}{ccc} \cos\psi \cos\theta & \cos\psi \sin\theta \sin\phi -\sin\psi \cos\phi & \cos\psi \sin\theta \cos\phi +\sin\psi \sin\phi \\ \sin\psi \cos\theta & \sin\psi \sin\theta \sin\phi +\cos\psi \cos\phi & \sin\psi \sin\theta \cos\phi -\cos\psi \sin\phi \\ -\sin\theta & \cos\theta \sin\phi & \cos\theta \cos\phi \end{array}\right],$$

(3) $$J_{2}\left(\eta \right)=\left[\begin{array}{ccc} 1 & \tan\theta \sin\phi & \tan\theta \cos\phi \\ 0 & \cos\phi & -\sin\phi \\ 0 & \sec\theta \sin\phi & \sec\theta \cos\phi \end{array}\right],$$

The motion law of the undulating fin is described in the fin coordinate system. Let the coordinates of any point on the fin surface in the $O_{f}X_{f}Y_{f}Z_{f}$ system be $P\left(x_{f},y_{f},z_{f}\right)$. When the initial phase of the left and right undulating fins is zero and the waveform is completely symmetrical, the undulation equations are as follows:

(4) $$\mathrm{Left}:\;\{\begin{array}{l} x_{f}=s,\\ y_{f}=r\cdot \cos\left[\theta_{m}\sin\left(\delta_{1}\cdot 2\pi f_{1}+\frac{2\pi }{\lambda }s\right)\right],\\ z_{f}=r\cdot \sin\left[\theta_{m}\sin\left(\delta_{1}\cdot 2\pi f_{1}+\frac{2\pi }{\lambda }s\right)\right], \end{array}$$

(5) $$\mathrm{Right}:\;\{\begin{array}{l} x_{f}=s,\\ y_{f}=r\cdot \cos\left[\theta_{m}\sin\left(\delta_{2}\cdot 2\pi f_{2}+\frac{2\pi }{\lambda }s\right)\right],\\ z_{f}=r\cdot \sin\left[\theta_{m}\sin\left(\delta_{2}\cdot 2\pi f_{2}+\frac{2\pi }{\lambda }s\right)\right], \end{array}$$

where *s* is the natural coordinate in the baseline direction of the undulating fin, *r* is the natural coordinate in the fin direction, *f*_1_ and *f*_2_ are the left and right undulating frequencies, respectively, λ is the wavelength, $\theta_{m}$ is the wave amplitude, and $\delta_{1}$ and $\delta_{2}$ represent the wave transmission direction. When $\delta_{i}=-1$, the wave is propagated in the −*x* direction; when $\delta_{i}=1$, the wave is propagated in the +*x* direction.

Let the origin of the left and right undulating fins in the local coordinate system be $O_{f1}\left(a,-b,0\right)$ and $O_{f2}\left(a,b,0\right)$, respectively. a and b are determined by the robot geometric shape. The angles between the undulating fin and the body are$\varphi_{1}$ and $\varphi_{2}$. According to the rotation relationship, the expression of the fin surface in the body coordinate system is as follows:

(6) $$\mathrm{Left}:\;\left[\begin{array}{c} x_{b}\\ y_{b}\\ z_{b} \end{array}\right]=\left[\begin{array}{ccc} 1 & 0 & 0\\ 0 & \cos\varphi_{1} & -\sin\varphi_{1}\\ 0 & \sin\varphi_{1} & \cos\varphi_{1} \end{array}\right]\left[\begin{array}{c} x_{f}\\ y_{f}\\ z_{f} \end{array}\right]+\left[\begin{array}{c} a\\ -b\\ 0 \end{array}\right],$$

(7) $$\mathrm{Right}:\;\left[\begin{array}{c} x_{b}\\ y_{b}\\ z_{b} \end{array}\right]=\left[\begin{array}{ccc} 1 & 0 & 0\\ 0 & \cos\varphi_{2} & -\sin\varphi_{2}\\ 0 & \sin\varphi_{2} & \cos\varphi_{2} \end{array}\right]\left[\begin{array}{c} x_{f}\\ y_{f}\\ z_{f} \end{array}\right]+\left[\begin{array}{c} a\\ b\\ 0 \end{array}\right].$$

Furthermore, the coordinates of point $P\left(x_{b},y_{b},z_{b}\right)$ within the geographical system $P\left(x_{e},y_{e},z_{e}\right)$ are determined as follows:

(8) $$\left[\begin{array}{c} x_{e}\\ y_{e}\\ z_{e} \end{array}\right]=J_{1}\left(\eta \right)\left[\begin{array}{c} x_{b}\\ y_{b}\\ z_{b} \end{array}\right]+\left[\begin{array}{c} X\\ Y\\ Z \end{array}\right].$$

In CFD simulation, the deformation motion of the undulating fins is defined by Equations (4) and (5). Through Equations (6)–(8), the coordinate of any points on the fin surface can be transformed between the global coordinate system, $O_{e}X_{e}Y_{e}Z_{e}$, and the fin coordinate system, $O_{f}X_{f}Y_{f}Z_{f}$. This kinematic relationship facilitates the definition and updating of dynamic mesh in fluid simulation analysis.

#### 3.2.2. Underwater Dynamic Model

The mass of the robot is defined as m, and the inertia tensor is $J\in R^{3\times 3}$. The moment of inertia of the spindle is *J_x_*, *J_y_* and *J_z_*. The three-dimensional force and moment of the robot underwater in the local robot coordinate system are expressed as $F={\left(F_{x},F_{y},F_{z}\right)}^{T}$ and $M={\left(M_{x},M_{y},M_{z}\right)}^{T}$. The centroid dynamic equation of the robot is as follows:

(9) $$\{\begin{array}{c} F=\frac{mdV}{dt}=mV^{\prime }+\omega \times mV,\\ M=\frac{d\omega }{dt}=J\omega^{\prime }+\omega \times J\omega . \end{array}$$

Substitute the velocity and angular velocity components; we can obtain the acceleration of each direction.

(10) $$\{\begin{array}{c} \dot{u}=vr-wq+\frac{F_{x}}{m}\\ \dot{v}=-ur+wp+\frac{F_{y}}{m}\\ \dot{w}=uq-vp+\frac{F_{z}}{m} \end{array},$$

(11) $$\{\begin{array}{c} \dot{p}=\frac{qr\left(J_{y}-J_{z}\right)}{J_{x}}+\frac{1}{J_{x}}M_{x}\\ \dot{q}=\frac{pr\left(J_{z}-J_{x}\right)}{J_{y}}+\frac{1}{J_{y}}M_{y}\\ \dot{r}=\frac{pq\left(J_{x}-J_{y}\right)}{J_{z}}+\frac{1}{J_{z}}M_{z} \end{array}.$$

When the force and the moment of the robot are solved, the acceleration of the robot can be obtained using Equations (10) and (11), and the linear and angular velocity can be obtained via the integral. Through rotation matrix transformation, the velocity, angular velocity, and displacement of the robot in the global coordinate system can be solved. Therefore, the hydrodynamic and motion performance of the robot in different swimming modes can be simulated.

### 3.3. Simulation Result

#### 3.3.1. Surge Motion

**Figure 8:**
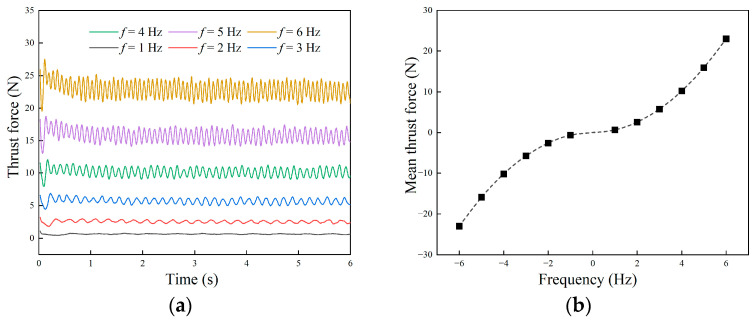
Simulated thrust force of undulating fins in fixed-body mode: (**a**) thrust force changing with time; (**b**) mean force at different frequencies.

When $f_{1}=f_{2}$ and $\delta_{1}=\delta_{2}$, the undulating fins on both sides propagate in the same direction with equal frequency. At this time, the two undulating fins produce forward or backward thrust at the same time, and the robot moves forward or backward linearly. Firstly, we simulated the in situ undulating motion of the robot under the condition of a fixed carrier. The change in the thrust force of the undulating fins under different frequencies is shown in Figure 8a, and the mean force is presented in Figure 8b. The propulsion force changed periodically with time, and the frequency of change was twice the frequency of the undulating fin. The higher the wave frequency, the greater the fluctuation amplitude and mean value of the propulsion force. The thrust generated by the undulating fin was proportional to the square of the wave frequency, and the direction of the thrust was opposite to the direction of the wave. Therefore, when the undulating fin transmitted the wave forward or backward, the generated force and motion were symmetrical.

**Figure 9:**
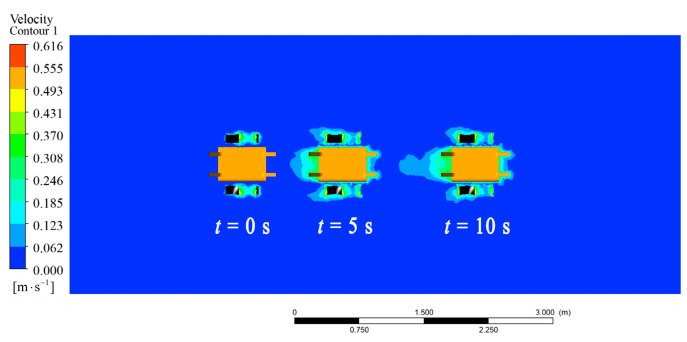
Velocity cloud image sequence of the amphibious robot in linear motion simulation.

**Figure 10:**
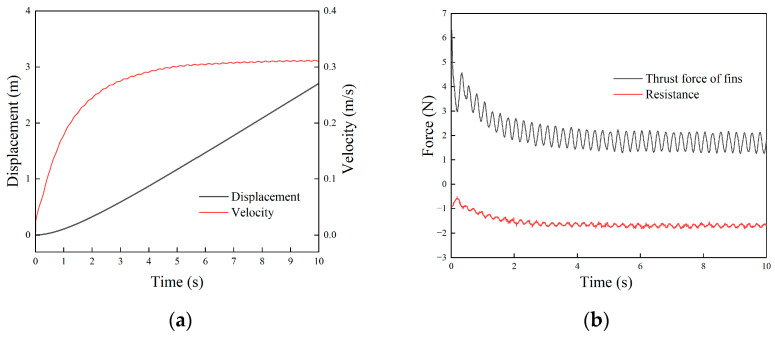
Simulation results of the amphibious robot in linear motion: (**a**) velocity and displacement; (**b**) thrust force and resistance.

Given $f_{1}=f_{2}=2\;\mathrm{Hz}$, the linear motion of the robot in the fluid was simulated. The animation video can be found in the Supplementary Materials, and the pressure cloud image sequence of the robot is shown in Figure 9. The different colors represented the velocity in the fluid field. The result showed that the joint force of the robot was forward, and the robot moved linearly without lateral and steering motion. The speed and displacement curves of the robot are shown in Figure 10a. The robot passed through a stage of acceleration, then reached the maximum speed and maintained a constant speed, and the steady-state average speed was about 0.31 m/s.

The thrust and fluid resistance suffered by the robot during movement are shown in Figure 10b. The results show that the thrust of the undulating fin was the largest in the initial stage, and then the thrust decreased gradually with the increase in the robot’s speed. At a stable stage, the thrust of the undulating fin also fluctuated around the average value.

According to the simulation result, the mean thrust force of the undulating fin can be simplified as follows:

(12) $$F_{T}=\mathrm{sign}\left(f\right)C_{T}{\left(\lambda f-V_{x}\right)}^{2},$$

where $C_{T}$ is the thrust coefficient, $V_{x}$ is the linear velocity, and sign(f) is the symbolic function.

The resistance of the robot can be expressed as follows:

(13) $$F_{D}=-\mathrm{sign}\left(V_{x}\right)\frac{1}{2}\rho SC_{D}V_{x}^{2},$$

where ρ is the fluid density, *S* is the cross-sectional area of the carrier, and $C_{D}$ is the resistance coefficient. In the steady stage, the resultant force is zero, which means $F_{T}+F_{D}=0$. Therefore, the steady-stage velocity is solved as follows:

(14) $$V_{x}=\frac{\lambda f}{1+\sqrt{\frac{\rho SC_{D}}{2C_{T}}}},$$

**Figure 11:**
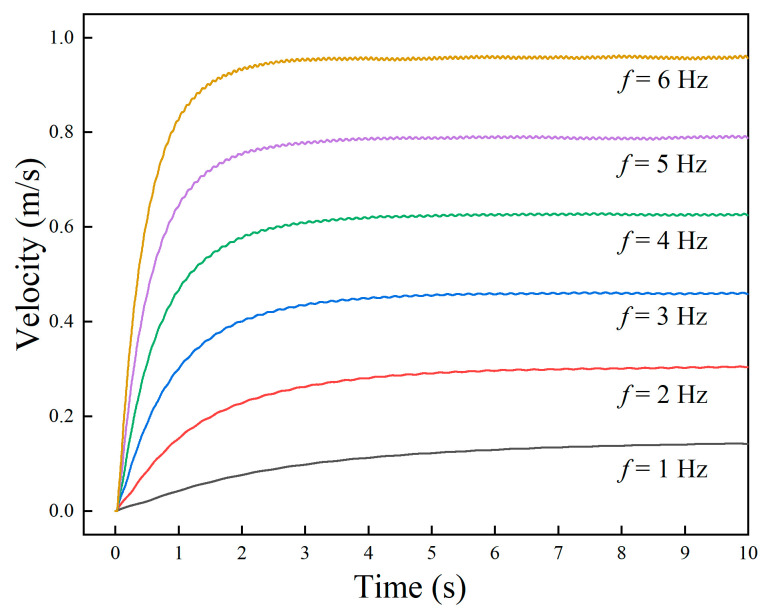
Propulsive velocity of the amphibious robot under different frequencies.

The propulsion speed of the robot at different frequencies was simulated, and the results are shown in Figure 11. The steady-state average speed of the robot increased linearly with the wave frequency. The higher the frequency, the greater the starting acceleration, and the shorter the time required to reach the maximum speed. The simulation result was consistent with the law of Equation (14).

#### 3.3.2. Steering Motion

**Figure 12:**
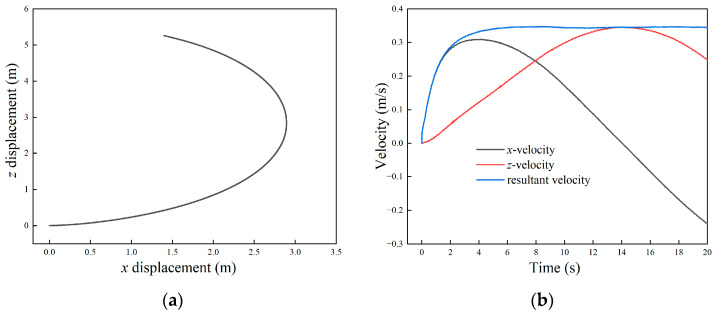
Velocity and displacement of the amphibious robot in steering motion simulation: (**a**) displacement in *x* and *z* direction; (**b**) velocity in *x* and *z* direction.

The motion performance of the robot in steering mode was simulated. Given the left and right frequencies of *f*_1_ = 2.5 Hz and *f*_2_ = 2 Hz, the robot was supposed to deflect to the right side while moving forward. The trajectory of the robot obtained via simulation is shown in Figure 12a. The robot maintained steering during the movement, and passed through an arc trajectory in the *xz* plane. The tangential of any point on the trajectory corresponds to the combined speed directions of the robot.

The sub-velocity in the *x* and *z* directions and the combined velocity of the robot are shown in Figure 12b. The *x*-velocity increased first and then decreased until it became negative. The velocity in the *z*-axis increased first and then decreased, corresponding to the two stages of the robot’s positive and negative motion in the *x* direction. When *t* = 14 s, the *x*-velocity was zero, and the corresponding *z*-velocity was the largest. The total velocity of the robot reached the maximum value at about 6 s, and then remained stable at about 0.35 m/s.

**Figure 13:**
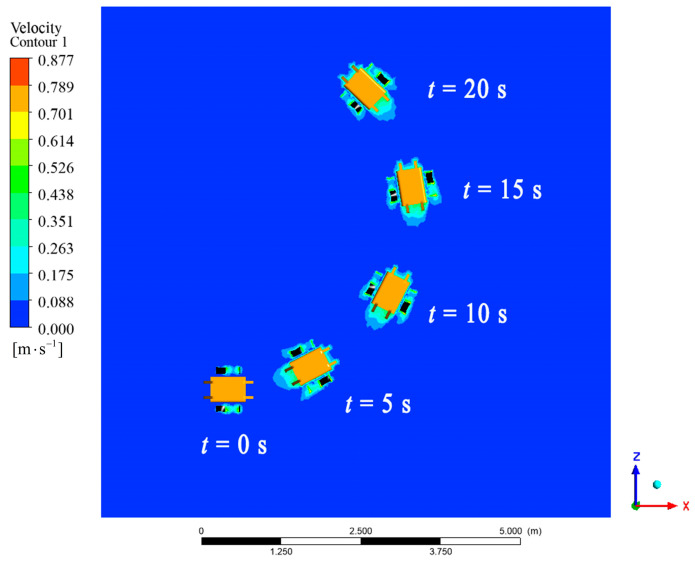
Velocity cloud image sequence of the robot in steering motion simulation.

The motion posture and spatial position of the robot are shown in Figure 13 (the animation video can be found in the Supplementary Materials). The result showed that the velocity of the flow field was small at the beginning. With the drive of the undulating fins, the velocity obtained by the robot from the fluid gradually increased and tended to be stable. Under the asymmetric waveform, the robot successfully realized the steering maneuver movement. The simulation results were consistent with the theoretical analysis.

#### 3.3.3. Rotation Motion

**Figure 14:**
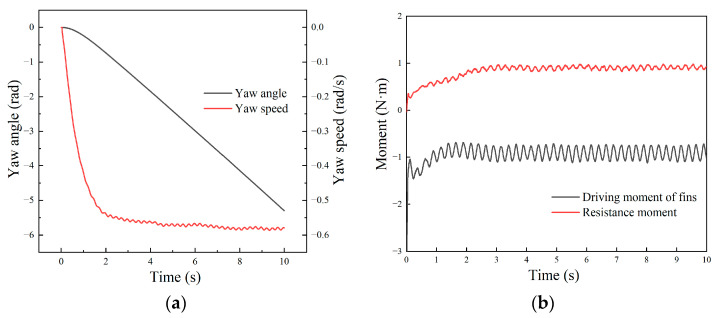
Motion and hydrodynamic moment of the robot in rotation motion simulation: (**a**) yaw angle and speed; (**b**) driven moment and resistance moment.

The in situ rotation mode is a special case of steering motion, in which case the wave of the left and right fins transmit in the opposite direction. The thrust forces cancel each other out and form a rotating torque around the center of the robot. The frequencies are given as $f_{1}=f_{2}=2$ Hz and $\delta_{1}=-\delta_{2}=1$, and the motion of the robot’s right turn in situ is simulated. The yaw angle and speed are shown in Figure 14a. The results show that the yaw angular velocity experienced two stages: starting acceleration and stable maintenance. The yaw angle gradually increased with time, and the oblique rate increased first and then remained unchanged, which corresponded to the regulation of angular velocity. The steady-state average yaw rate was about −0.58 rad/s.

During the rotation motion, the relationship between the driving moment provided by the undulating fins and the fluid resistance moment on the carrier over time is shown in Figure 14b. The results showed that the driving moment was largest in the initial stage, then gradually decreased and eventually fluctuated around the stable value. The change regulation of the resistance moment was opposite. The resistance increased with the rotation speed. During the stable stage, the driving moment and the resistance were balanced, and the robot conducted uniform steering motion. We can find that the rotation motion presented similar regulations with the linear motion, which corresponded to the results of the dynamic analysis of the undulating fins and the theoretical analysis of the fluid resistance.

**Figure 15:**
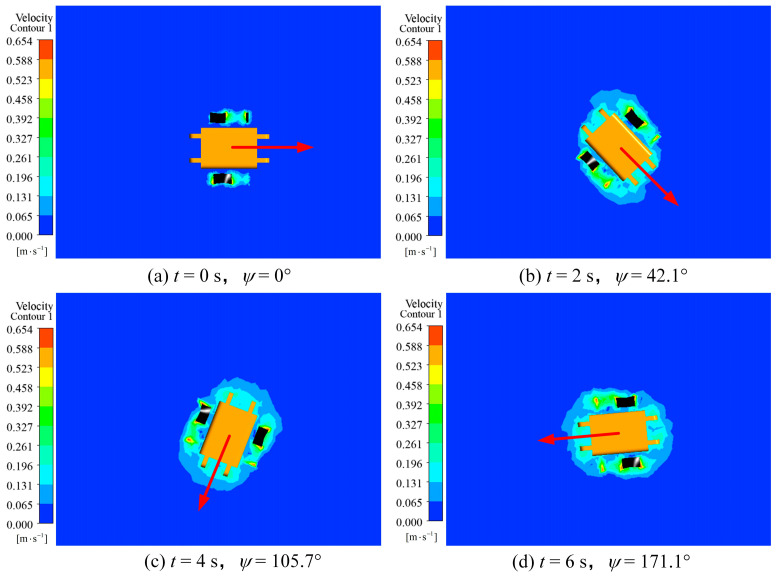
Velocity cloud image sequence of the robot in the rotation motion simulation: (**a**) *t* = 0 s; (**b**) *t* = 2 s; (**c**) *t* = 4 s; (**d**) *t* = 6 s.

The velocity cloud image sequence of the rotation motion process is shown in Figure 15, which showed that the robot realized continuous rotation motion around the center of mass, and the position of the robot did not change, indicating that the robot only rotated in situ without translational motion. The animation video can be found in the Supplementary Materials.

**Figure 16:**
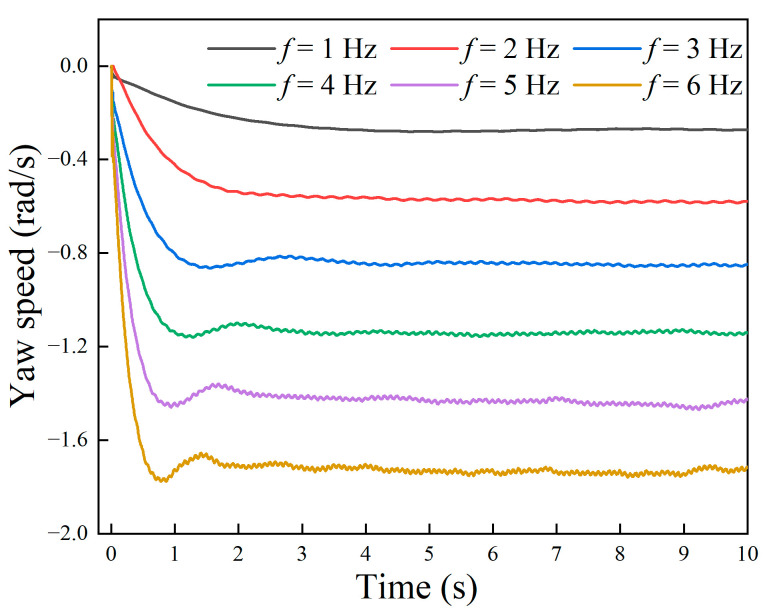
Simulated underwater rotation speed of the robot under different frequencies.

Further, the angular velocity of the robot at different undulating frequencies was simulated, and the results are shown in Figure 16. The stable angular velocity of the robot increased linearly with the wave frequency. The higher the frequency, the greater the starting acceleration, and the shorter the time required to reach the maximum angular velocity. When the frequency was 6 Hz, the maximum rotation angular velocity reached −1.75 rad/s.

## 4. Experiment Validation

### 4.1. Robot Prototype

**Figure 17:**
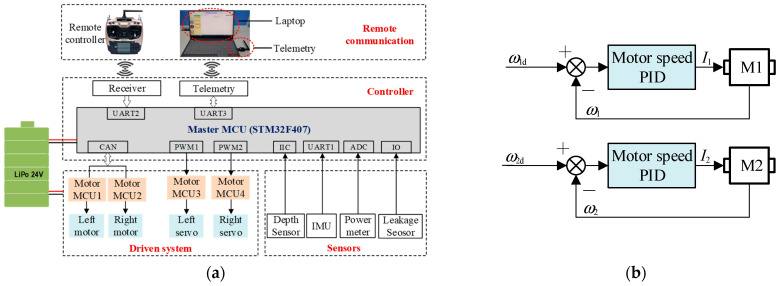
Hardware and control algorithms: (**a**) hardware scheme; (**b**) PID controller.

To verify the robot’s hydrodynamic performance in the CFD simulation, a robot prototype was developed for physical experiments. We integrated hardware circuits and control software for the robot. The hardware scheme is shown in Figure 17a. The master control board was based on an STM32F407 microchip (ST, Geneva, Switzerland), featuring an abundant peripheral interface. The control software was programmed on the Keil MDK5.14 platform. The control tasks included motor control, sensor data acquisition, wireless communication, and human–computer interaction. The drive motors adopted M3508 (DJI, Shenzhen, China) and the motor MCU communicated with the control board through the CAN bus. The two steering engines were controlled by a PWM signal, which could change the deviation angle between the undulating fin and the robot body. The robot was equipped with various sensors, including water depth sensors, inertial measurement units (IMUs), power meters, and leakage detection sensors. The sensor data were read through IIC, UART and other interfaces. The robot adopted wireless communication. We assigned two serial ports to connect the receiver and the data telemetry, respectively. The robot could be controlled either by handheld remote control (RadioLink, Shenzhen, China) or by input commands from a portable computer. The former method was ideal for field operation, while the latter was suitable for data monitoring and program debugging. The robot was powered by a lithium battery (24 V, 6000 mAh).

At the present stage, we primarily focused on the speed control of the robot, with the control principle illustrated in Figure 17b. The control board received the frequency command for the undulating fins and translated them into the desired angular velocity for each motor. Subsequently, a PID control algorithm was applied to regulate the motor speed. The actual speeds were transmitted by the motor driver via the CAN bus at a frequency of 1000 Hz. The master MCU then compared the target speed with the measured speed, executing separate PID controls for each motor and calculating the control current to be sent to the motor driver through the CAN bus. By managing the speeds of motors on either side, the robot could effectively conduct multimodal motion both on land and underwater.

### 4.2. Underwater Maneuverability

#### 4.2.1. Underwater Linear Motion

**Figure 18:**
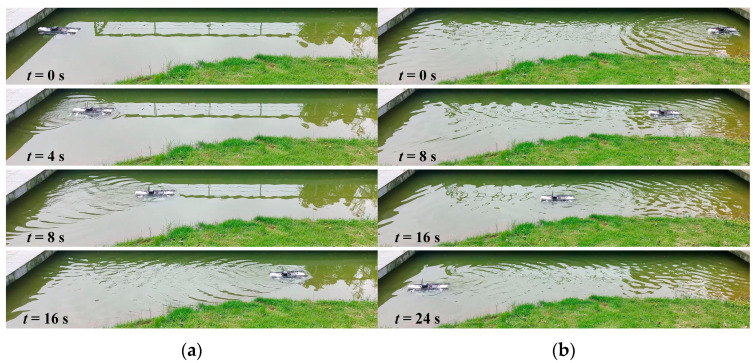
Experiment results of underwater linear forward and backward motion at 3 Hz: (**a**) forward motion; (**b**) backward motion.

To verify the robot’s hydrodynamic and motion performance in the CFD simulation, experiments were conducted in outdoor aquatic environments based on the robot prototype. The underwater experiments video can be found in the Supplementary Materials. The linear motion experiment image sequence of the robot when the undulating frequency was 3 Hz is presented in Figure 18. The results prove that by controlling the wave direction of the undulating fins, the robot can generate vector thrust, thereby flexibly achieving forward and backward movements. In addition, the robot can maintain a relatively fixed heading angle.

**Figure 19:**
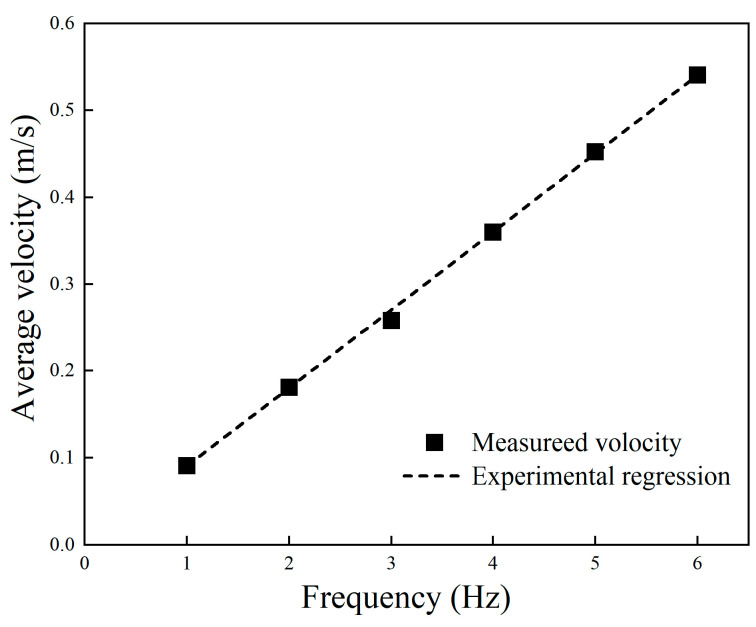
Experiment result of underwater propulsive velocity at different frequencies.

The maximum velocity that the robot could achieve at different undulating frequencies was tested, and the result is shown in Figure 19. The squares were the measured velocity and the dotted line was the regression model. The curve illustrates that the maximum velocity was improved with the increase of in wave frequency, and is approximately linear to the frequency, which is consistent with the simulation results. When the frequency was 6 Hz, the speed of the robot was 0.54 m/s. As the length of the robot was 0.81 m, the maximum speed was 0.67 BL/s.

#### 4.2.2. Underwater Steering and Rotation Motion

**Figure 20:**
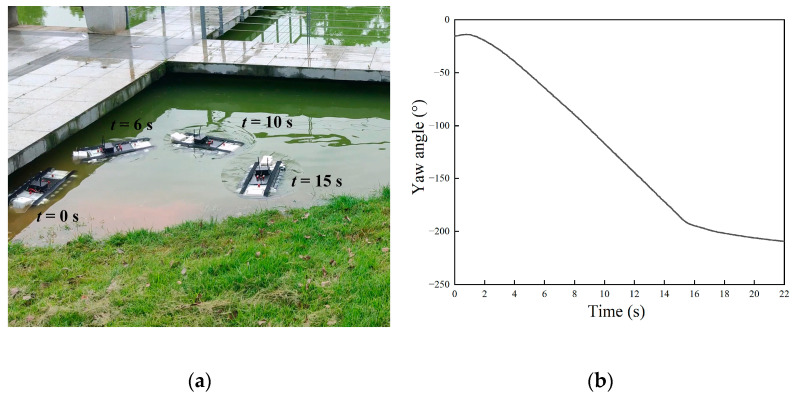
Experiment results of underwater steering motion: (**a**) motion images; (**b**) yaw angle.

Given the undulating frequencies of *f*_1_ = 3 Hz and *f*_2_ = 2 Hz, the motion image of the robot is shown in Figure 20a, and the change in the yaw angle is shown in Figure 20b. The experimental phenomenon was consistent with the simulation analysis. The robot continuously deflected to the right side while moving forward. After about 4 s, the angular speed remained constant at about 13.5°/s. When the stop command was issued at 15 s, the robot continued to deflect at a certain angle due to inertia.

**Figure 21:**
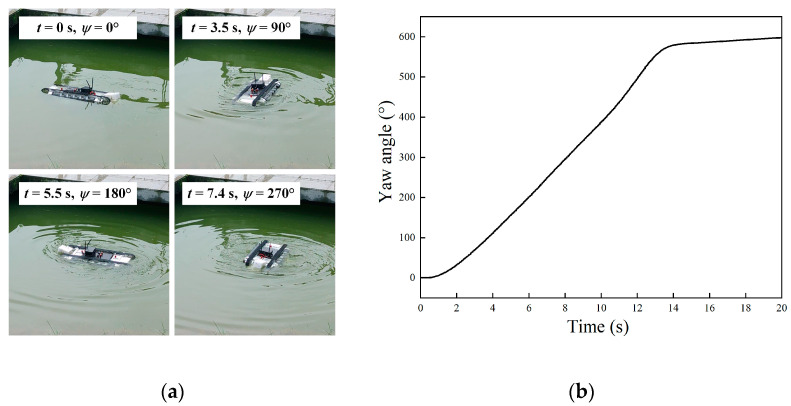
Experiment results of underwater rotation motion. (**a**) motion images; (**b**) yaw angle.

When the undulating frequencies were set as *f*_1_ = *f*_2_ = 3 Hz and the directions were opposite, the robot conducted in situ rotation locomotion. The motion image of the robot is shown in Figure 21a, and the yaw angle is shown in Figure 21b. The results indicated that the robot performed a continuous left turn movement and the position of the center of gravity remained unchanged. The robot initially accelerated until the driving moment was balanced with the resistance moment, at which time the robot attained the maximum speed. In the steady stage, the robot subsequently rotated at a constant speed of about 45°/s.

**Figure 22:**
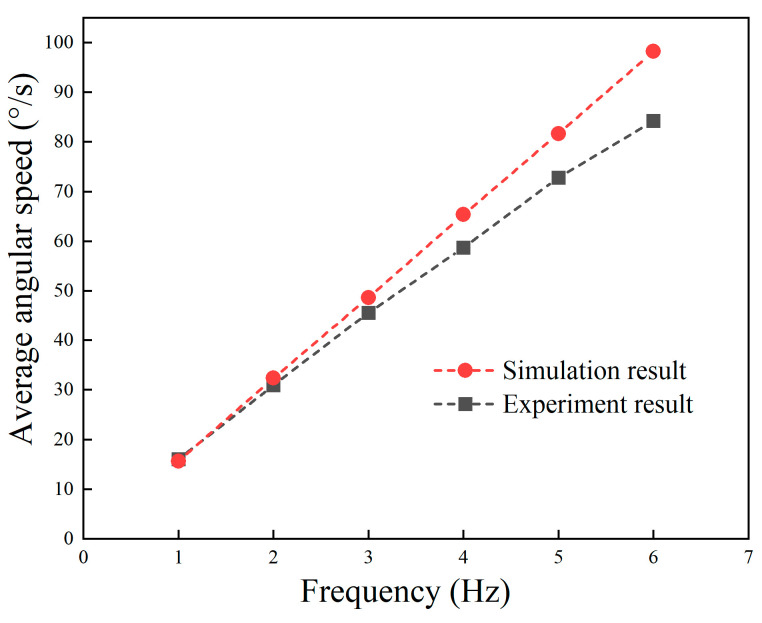
Experiment result of underwater rotation speed at different frequencies.

Further, the maximum rotation speed that the robot could achieve under different undulating frequencies was measured, and the result is shown in Figure 22. The average rotation speed of the robot was approximately linear to the increase in frequency, which is consistent with the theoretical analysis and fluid simulation result. When the undulating frequency was 6 Hz, the rotation speed of the robot could reach 84°/s.

According to the experiment results, both the linear and rotation speed were relatively slower than those in the simulation results. This may be because the resistance force and moment of the real prototype were larger than those in the simulation model. The shape of the robot prototype was not continuous, while the simulation model was simplified for the convenience of grid division and calculation accuracy. Furthermore, the waveform of the mechanical undulating fin was fitted by the finite fin rays, and this fit was not perfect compared with that of the theoretical model in the simulation environment, thus leading to smaller driving force.

### 4.3. Terrestrial Maneuverability

#### 4.3.1. Terrestrial Linear Motion

**Figure 23:**
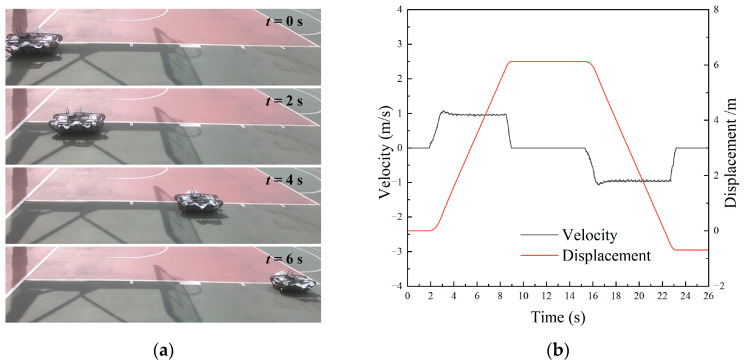
Experiment results of land linear motion: (**a**) motion images; (**b**) velocity and displacement.

Through the previous analysis, the robot has the same control strategy on land and underwater, and we tested the robot’s motion performance on land. The terrestrial locomotion video can be found in the Supplementary Materials. Setting the tracks’ speed as *f*_1_ = *f*_2_ = 3 Hz ($\omega_{1}\;$=$\;\omega_{2}=$180 r/min), and given that the directions were the same, the forward motion images of the robot are shown in Figure 23a.

We utilize track speed and yaw angle as odometric parameters and calculate the robot’s velocity and planar displacement. The centroid velocity of the robot is computed as follows:

(15) $$V=\left(\omega_{1}+\omega_{2}\right)R/2,$$

where *R* is the radius of the driven wheel.

By decomposing the displacement in each time step, the total displacement of the robot at time *T* in the *x*-axis and *y*-axis directions can be obtained:

(16) $$\{\begin{array}{c} ds=Vdt,\\ dx=ds\cos\psi \Rightarrow S_{x}=\int_{0}^{T}dx,\\ dy=ds\sin\psi \Rightarrow S_{y}=\int_{0}^{T}dy, \end{array}$$

The velocity and displacement curves in linear motion are shown in Figure 23b. The initial phase of the robot experienced a period of acceleration until the motor reached the preset speed. Subsequently, the robot maintained a constant speed in a straight line. When commanded to stop, the robot braked promptly. After that, upon receiving the backward command, the robot moved backward to the starting position in a straight line at the same frequency.

**Figure 24:**
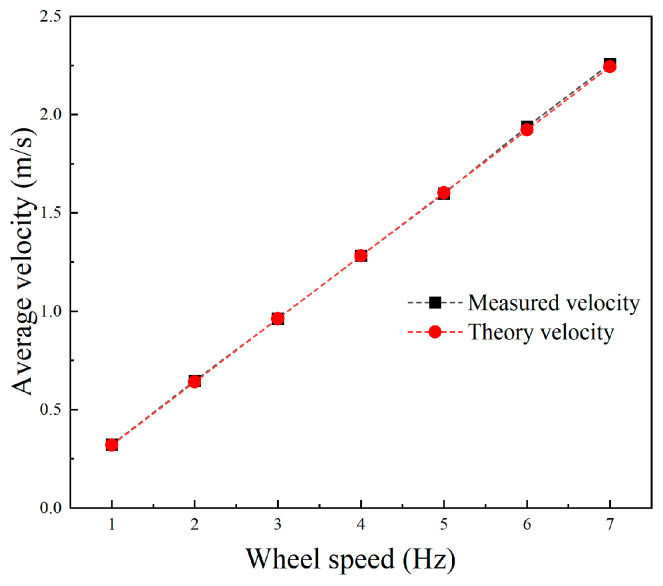
Experimental propulsion velocity on land at different wheel speeds.

Given different speeds of the track, the average speed of the robot walking on land was tested, and the results are shown in Figure 24. The black line is the speed measured by the external laser distance meter, and the red line is the theoretical speed calculated by the wheel odometer. The results showed that the speed of the robot was proportional to the motor speed, and the test speed was almost consistent with the theoretical speed, indicating that the robot had no slip with the ground and the motor control accuracy was excellent. Using the speed of motor feedback as a vehicle odometer would have had satisfactory accuracy. When the driving frequency was 7 Hz (the track speed was 420 r/min), the propulsion speed of the robot reached 2.26 m/s, corresponding to 2.8 BL/s.

#### 4.3.2. Terrestrial Steering and Rotation Motion

**Figure 25:**
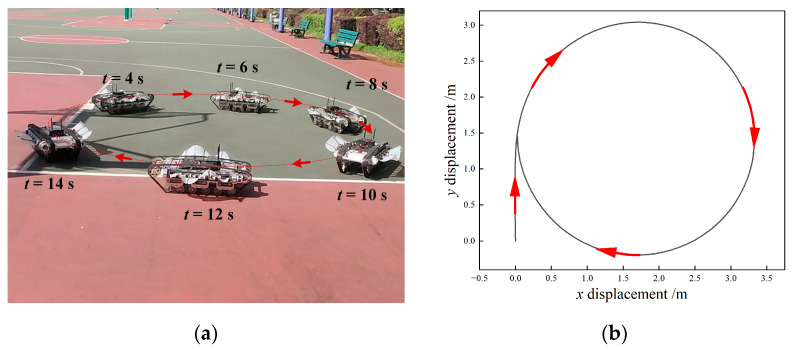
Experiment results of land steering motion. (**a**) motion images; (**b**) displacement.

The yaw motion on land was identical to the underwater case. When the left and right tracks rotated at different speeds, the robot would deviate to the lower-velocity side due to kinematic constraints. The steering locomotion experiment result is shown in Figure 25a, in which case we set *f*_1_ = 3 Hz, *f*_2_ = 2 Hz. The planar displacement of the robot is presented in Figure 25b, where the black curve represented the continuous position and the arrows indicated the velocity direction. The experimental result was consistent with the theoretical analysis. The robot conducted continuous right steering. The trajectory of the robot in the steady-state process was approximately a circle, which meant that there existed a fixed instantaneous center of rotation. Similarly, when the speed on the right side was larger, the robot carried out a left turn movement. By regulating the speeds of the left and right tracks, the robot could be controlled to rotate around any point.

**Figure 26:**
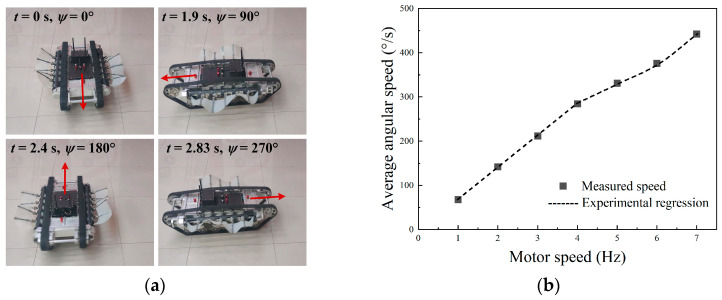
Experiment results of land rotation motion: (**a**) motion images at *f* = 2 Hz; (**b**) angular speed at different frequencies.

When the left and right tracks rotate in the opposite direction with the same frequency, the instantaneous rotation center falls on the geometric center of the robot, so the robot will rotate around itself. Given the left and right tracks frequencies of *f*_1_ = *f*_2_ = 3 Hz in opposite directions, the image sequence of the robot is shown in Figure 26a. The results showed that the position of the robot center was almost unchanged during the movement. As the motor started to accelerate, the yaw angular velocity gradually increased and finally stabilized at the maximum angular velocity. The rotation speed of the robot was 212°/s in the steady state.

We tested the angular velocity of the robot at various differential frequencies, and the result is presented in Figure 26b. The square dots were the measured angular speed and the line was the regression model. As the track speed escalated, the robot’s angular speed was significantly enhanced. When the track frequency was 7 Hz, the maximum speed reached 442°/s. The result also indicated that the angular speed was not linearly correlated to the track speed. However, within the range of 1–4 Hz, it could be approximately assumed that the steering speed was proportional to the frequency, facilitating robot motion analysis and trajectory control at medium and low speeds.

## 5. Conclusions

Conventional robots face challenges such as substantial size, excessive noise, limited adaptability to environments, and poor cross-media motion ability. To enhance their capability in executing tasks within complex and unstructured environments, it is essential to draw inspiration from the wide array of species that inhabit oceans, lakes, and river banks. Deriving inspiration from nature leads to a bioinspired system, which holds the potential to solve the technical challenges faced by traditional structures and actuators in existing robots. In this paper, we present a novel bionic amphibious robot that takes inspiration from stingrays. The robot was equipped with a pair of bionic undulating fins that propelled it in the water as efficiently as a fish. Furthermore, it also exhibited exceptional maneuverability and speed on land via the compound drive of tracks. We employed fluid simulation technology to accurately replicate the multi-modal motion of the robot underwater, thereby establishing a theoretical foundation and providing feasibility validation for subsequent prototype development. Through the prototype experiment, the performance of the robot’s amphibious locomotion was demonstrated, which verified the robot’s advancements in self-adaptive drive and self-organization control. The main conclusions are as follows.

(1) A biologically inspired amphibious robot was designed. Through a compound drive mechanism, a pair of bionic undulating fins and a pair of tracks were parallelly equipped on the robot, which were responsible for efficient locomotion underwater and on land, respectively. Sharing a same driving source enabled autonomous switching of the track and the fin, along with a unified motion control strategy both on land and underwater. Therefore, the robot did not need to judge in which kind of environment it was, as the robot’s locomotion and control principles such as forward, backward, and turning were the same in various environments.
(2) Based on the kinematics and dynamics model of the robot as well as the motion equation of the undulating fins, the hydrodynamic and motion performance of the robot under linear motion, steering motion, and in situ rotation motion were simulated using dynamic mesh method. The simulation results showed that the robot could generate vector thrust through the wave-like motion of the undulating fins. Both the linear swimming speed and the turning speed achieved by the robot were proportional to the wave frequency.
(3) The prototype experiment validated the amphibious motion performance of the robot. Using the same motion control strategy, the robot was capable of achieving unified forward and backward, steering, and in-place rotation locomotion both on land and underwater. The maximum linear speed and steering speed on land were 2.26 m/s (2.79 BL/s) and 442°/s, respectively. The maximum linear speed and steering speed underwater were 0.54 m/s (0.67 BL/s) and 84°/s, respectively.

Due to its remarkable mobility and high speed in both aquatic and terrestrial environments, the proposed amphibious robot will have broad application prospects, such as search and rescue, field reconnaissance, resource exploration, and environmental monitoring. In the future, we will focus on developing the robot’s accurate position control and path planning functions.

## Figures and Tables

**Table 1:** CFD simulation parameters.

Parameter type	Parameters/Unit	Value
Robot body	Body length, *L*/m	0.775
	Body width, *W*/m	0.404
	Body height, *H*/m	0.22
Undulating fin	Fin length, *L_f_*/m	0.38
	Fin width, *h*/m	0.15
	Fin thickness, *t*/m	0
	$\mathrm{Wave}\;\mathrm{amplitude},\;\theta_{m}$/°	19.8
Control parameters	Left fin frequency, *f*_1_/Hz	0–6
	Right fin frequency, *f*_2_/Hz	0–6
Dynamic parameters	Weight, *m*/kg	17
	Rotational inertia, *J_x__x_*/kg·m²	0.132
	Rotational inertia, *J_y__y_*/kg·m²	0.936
	Rotational inertia, *J_z__z_*/kg·m²	0.863

## Data Availability

The data presented in this study are available on request from the corresponding author upon reasonable request.

## Supplementary Materials

The following supporting information can be downloaded at https://www.mdpi.com/article/10.3390/biomimetics9100580/s1: Video S1: simulation_animation.mp4. Video S2: experiment_video.mp4.
